# Pilot study of minimum occlusive force of vascular clamps on arterial vessels in rats

**DOI:** 10.1038/s41598-021-84346-y

**Published:** 2021-03-15

**Authors:** Rui Sun, Lizhi Ren, Zepeng Zhang, Xiaofen Wu, Qianqian Wang, Sui Zhang, Xiaowen Yang

**Affiliations:** 1 Department of Oral and Maxillofacial Surgery, Shanxi Provincial People’s Hospital, Shanxi Medical University, Taiyuan, 030012 Shanxi China; 2 Shanxi Medical University and Hospital of Stomatology, Taiyuan, China; 3 Shanxi Provincial People’s Hospital, Shanxi Medical University, Taiyuan, China

**Keywords:** Computational biology and bioinformatics, Medical research, Mathematics and computing

## Abstract

Our aims were to determine the accuracy of an improved formula for determining the minimum occlusive force (MOF) of a vascular clamp on rats’ abdominal aortas, compare our findings with the calculated theoretical MOF, and provide reference data for clinical research and development of medical instruments that cause minimal damage. We created a vessel closure model and developed a formula for calculating the theoretical MOF of arterial vessels when they are occluded. This formula utilises the blood pressure in the blood vessel, its diameter, and the width of the vascular clamp. We then measured the actual MOF in 24 rat abdominal aortic segments with different diameters and different blood pressures and compared the theoretical and actual MOFs. Analysis of the experimental data showed a probability of 0.315, which means that, under the condition of normal distribution, the difference between the theoretical and actual MOF is not significant at the α = 0.05 level. Thus, the actual measured MOF tended to be consistent with the theoretical MOF calculated by the formula we developed. The improved formula will provide a reference for clinical research and development of medical instruments that cause minimal injury, thus contributing to the development of microsurgery.

## Introduction

Creating vascular anastomoses in microsurgery and cardiovascular surgery requires stopping blood flow in blood vessels by occluding them with vascular clamps^1^. The use of vascular clamps in contemporary microsurgery can facilitate completion of the procedure. However, vascular clamps may damage blood vessels, an issue that is the subject of ongoing research^2–10^. In 1979, Dujovny and others were the first to propose the concept of minimum occlusive force (MOF), this being defined as the MOF created by a vascular clamp that completely blocks blood flow while causing minimal damage to the intima. A computer-generated mathematical prediction of the MOF involves three factors, namely blood vessel diameter, blood pressure, and leaf contact area^11^. In 1997, Trobec et al. found that the occlusive force on the vascular wall (the force on the vascular wall created by a vascular clamp) can directly result in vascular intimal damage^12^.

The aims of the present study were to improve the formula for calculating the MOF on an artery by analysing a stress model of the artery and measuring the MOF on that artery, thus providing a reference for clinical research and development of medical instruments that cause minimal injury. In turn, this could facilitate manufacture and use of vascular clamps that can safely prevent blood flow while inflicting minimal or no damage. Clinicians could then assess their patients’ basic characteristics and select appropriate vascular clamps to achieve minimal vascular damage.

## Methods

All experimental methods were performed in accordance with the relevant guidelines and regulations and conformed with the institutional guidelines approved by the Chinese Association of Laboratory Animal Care. All experimental protocols were approved by the Institutional Animal Care and Use Committee and the Animal Experimental Ethics Committee of Shanxi Provincial People’s Hospital, Shanxi Medical University Taiyuan, China approved the study.

### Experimental subjects and equipment

Forty-eight Sprague Dawley (SD) male rats weighing 280 g to 320 g were used in this study. The following equipment was also used: a biosignal acquisition system monitor (Calvin Biotechnology, Nanjing, China), microvascular clamp (Xinhua Surgical Instruments, Shandong, China), electronic digital callipers (Manette, Ellrich, Germany), tension meter (Desk, Eisenach, Germany), heparin sodium (Solarbio, Beijing, China), pentobarbital sodium (Sigma, San Francisco, CA, USA), and atropine (Borthayre, Wuhan, China).

### Anaesthesia and surgical procedures

**Figure 1:**
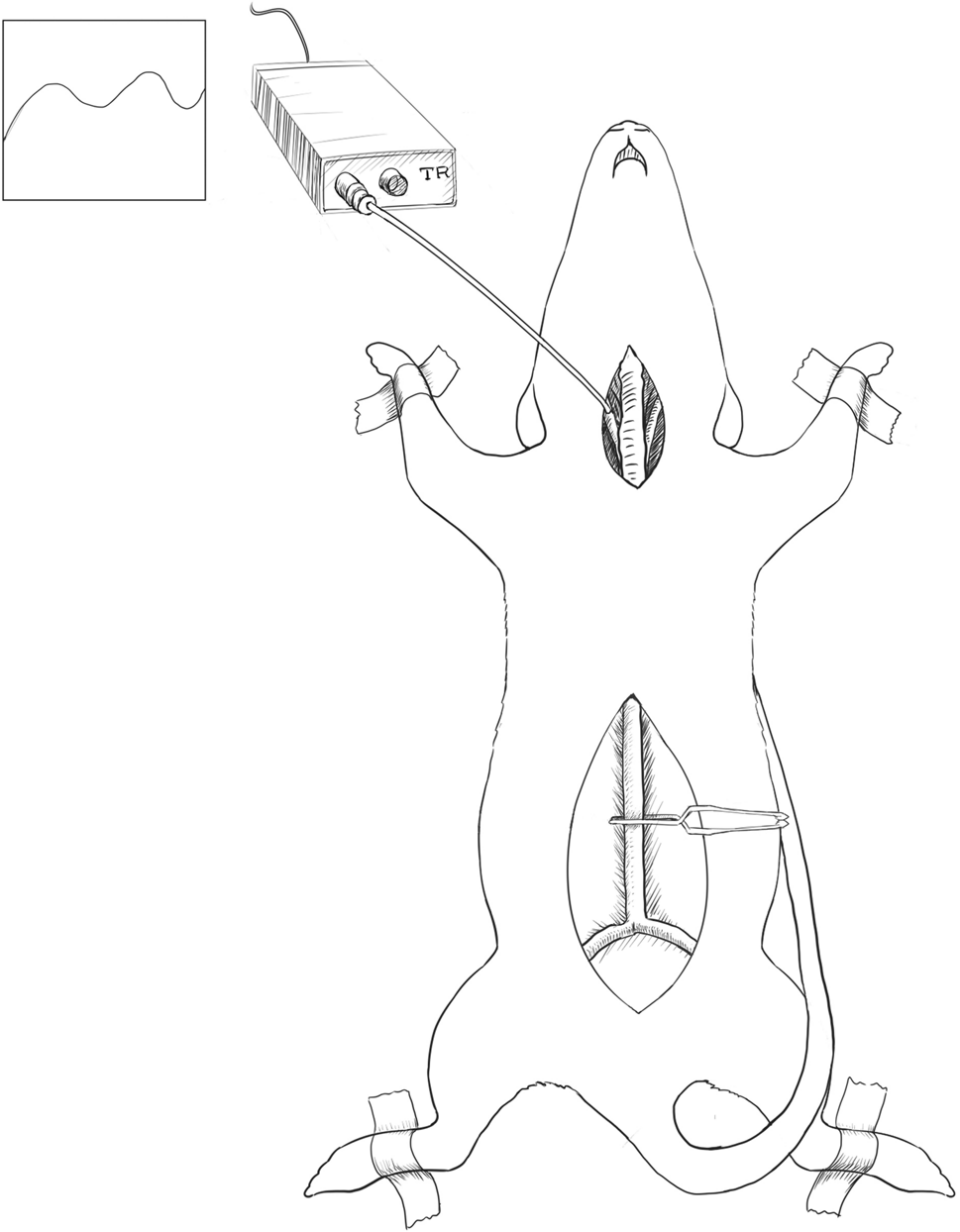
Schematic Diagram of Animal Experimental Surgery. Under general anaesthesia, the rats were placed in a supine position, the right internal carotid artery exposed, the internal carotid artery intubated and filled with heparin to prevent blood coagulation, and the other end of the catheter connected to the monitor of a biological signal acquisition system to detect ambulatory blood pressure. Relevant variables (ambulatory blood pressure, vessel diameter, vessel clamp width) and MOF were then measured. (Adobe Photoshop: 21.0.1 20,191,106.r.47 2019/11/06:3152b481f18 × 64 https://www.adobe.com/cn/products/photoshop.html).

All rats were anesthetized with pentobarbital sodium (40 mg/kg intraperitoneally) and 0.4 mg atropine subcutaneously. Throughout the study, all animals received humane care. Under general anaesthesia, the rats were placed in a supine position, the right internal carotid artery exposed, the internal carotid artery intubated and filled with heparin to prevent blood coagulation, and the other end of the catheter connected to the monitor of a biological signal acquisition system to detect ambulatory blood pressure. Relevant variables (ambulatory blood pressure, vessel diameter, vessel clamp width) and MOF were then measured, as shown in Fig. 1.

Still under general anaesthesia, the rats’ abdominal cavities were opened in layers directly caudad to the xiphoid process, the abdominal aorta was exposed and separated, and the diameter of the abdominal aorta was measured three times and averaged. Subsequently, the measured segment of the abdominal aorta was removed and the diameter of the empty segment of aorta was measured to obtain corresponding data and check the accuracy of the in vivo measurements.

### Mathematical analysis of vascular closure model

**Figure 2:**
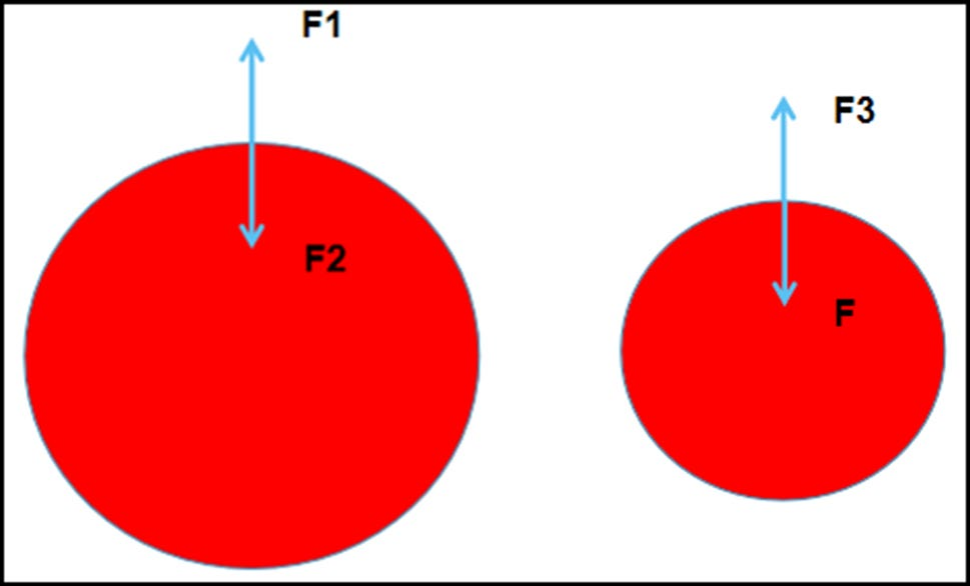
Schematic Diagram of Vascular Stress Analysis. We assume that a downward force F of magnitude F1 is exerted on the blood vessel wall and that this is offset by lateral pressure generated by blood on the blood vessel wall (F2), which deforms the blood vessel and results in it regaining its initial diameter. The diameter of the blood vessel is D at this moment. We can analyse the current state repeatedly and regard it as an ideal physical model provided that we ignore the local influence of other factors operating in the rats. At this time, the blood vessel is in a stress-free state without deformation. The forces acting on it are the downward force F, which we apply externally artificially, and the internal pressure of blood on the blood vessel wall with the diameter (d) at this moment. We term this F3 in the physical model. In this ideal model, the forces of F and F3 are also equal in theory. (Adobe Photoshop: 21.0.1 20,191,106.r.47 2019/11/06:3152b481f18 × 64 https://www.adobe.com/cn/products/photoshop.html).

To facilitate our analysis, a physical model of vessel closure was built, as shown in Fig. 2. First, blood is a non-Newtonian fluid^13^, which means that complicated finite element analysis is required to investigate it. However, the fluid pressure calculation method in this paper assumes that it is Newtonian fluid.

Newton’s laws of motion state that effects are relative and the forces on stationary objects are balanced. Thus, we can regard a blood vessel filled with blood as a static object. The blood in a blood vessel produces a pressure on the vessel wall, termed blood pressure, which is expressed by F1 in the physical model. The formula for calculating the pressure is F = P × S; this is thus the product of the pressure per unit area and the contact area. The influencing factors include vessel diameter (D), blood pressure (BP) and vessel clamp width (W). These data are required for the following equation.

1 $${\text{F}}1 = 1/2 \times\uppi \times {\text{D}} \times {\text{W}} \times {\text{BP}}$$

From the lumen to the outside, vessel walls have inner, middle, and outer membranes that contain numerous collagen and elastic fibres^14^, which have a certain degree of elasticity. When a blood vessel goes from this static state, the filled blood vessel wall expands, which forms a resilience force in the blood vessel wall that is represented by F2 in the physical model. Some scholars have performed three-dimensional finite element analyses of vessel walls^15^ and undertaken corresponding research on the elastic modulus of some vessels^16^. The term “elastic modulus” (E) refers to the stress required by a material to produce unit elastic deformation under the action of an external force. The greater its value, the greater the stress that causes the material to undergo certain elastic deformation; that is, the greater the stiffness of the material, the smaller the elastic deformation under a certain stress. The rebound force per unit area is equal to the product of the elastic modulus and strain. In the present study, the experimental blood vessels were the abdominal aortas of SD rats, the elastic modulus of which is approximately 0.0603 ± 0.0044 N/mm^2^^16^. The strain (ε) is the ratio of the deformation when the blood vessel is not congested to when it is filled; this can be obtained by measurement and calculation as follows:

2 $${\text{F}}2 = {\text{E}} \times\upvarepsilon \times 1/2 \times\uppi \times {\text{D}} \times {\text{W}}.$$

Theoretically, the forces acting on F1 and F2 are equal and we can determine whether this is true. At this point, we assume that a downward force F of magnitude F1 is exerted on the blood vessel wall and that this is offset by lateral pressure generated by blood on the blood vessel wall (F2), which deforms the blood vessel and results in it regaining its initial diameter. As shown in Fig. 2, the diameter of the blood vessel is D at this moment. We can analyse the current state repeatedly and regard it as an ideal physical model provided that we ignore the local influence of other factors operating in the rats. At this time, the blood vessel is in a stress-free state without deformation. The forces acting on it are the downward force F, which we apply externally artificially, and the internal pressure of blood on the blood vessel wall with the diameter (d) at this moment. We term this F3 in the physical model. However, the local blood pressure may also change because of changes in the local vessel diameter as follows:

3 $${\text{F}}3 = 1/2 \times\uppi \times {\text{d}} \times {\text{W}} \times {\text{BP}}^{\prime } .$$

In this ideal model, the forces of F and F3 are also equal in theory. The last that requires mention is the rebound force F4 generated by deformation of the two layers of blood vessel wall by the pressure resulting from complete closure of a blood vessel. This is calculated as for F2 as follows:

4 $${\text{F}}4 = {\text{E}} \times\upvarepsilon ^{\prime } \times 1/2 \times\uppi \times {\text{d}} \times {\text{W}}.$$

### Determination of minimum occlusive force in vivo

**Figure 3:**
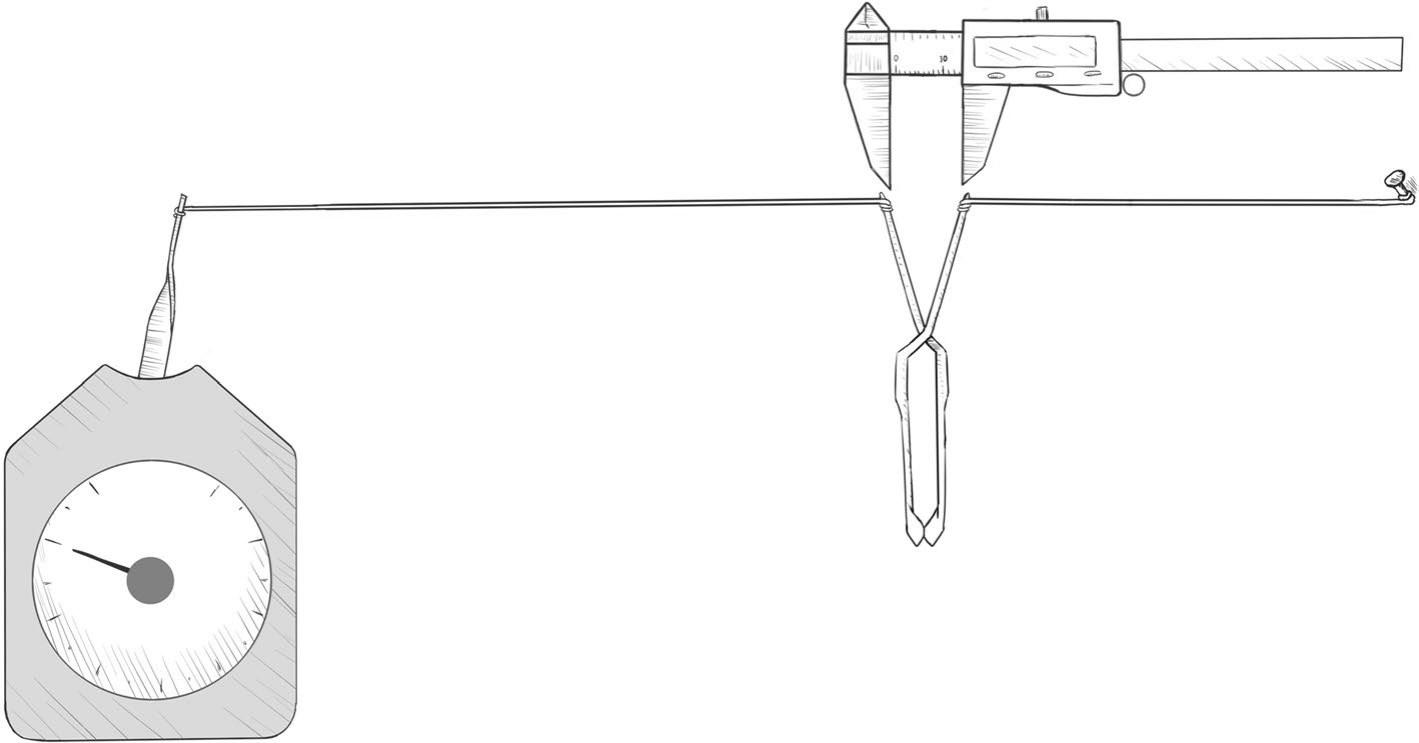
Schematic Diagram of Device for Measuring Occlusive Force of Blood Vessel Clamp. One third of each of the blades of the blood clamp was fixed with 4–0 silk threads, one side being fixed to the dynamometer sensing rod, and the other to the experiment table. The vascular clamp was then slowly pulled apart until the blade opening width was the same as the measured vascular diameter, and the required force, namely the occlusive force of the vascular clamp at the time, was recorded. (Adobe Photoshop: 21.0.1 20,191,106.r.47 2019/11/06:3152b481f18 × 64 https://www.adobe.com/cn/products/photoshop.html).

The MOF can be measured in vivo by using a German (desk) dial dynamometer, the minimum unit of which is accurate to 0.2 g. We did not use an electronic sensor dynamometer system in this study because the forces are relatively small and the sensitivity of the electronic components would have had a major influence on the experimental results. Although dial dynamometers also cause artificial errors, these errors can be controlled for by multiple measurements. One third of each of the blades of the blood clamp was fixed with 4–0 silk threads, one side being fixed to the dynamometer sensing rod, and the other to the experiment table. The vascular clamp was then slowly pulled apart until the blade opening width was the same as the measured vascular diameter, and the required force, namely the occlusive force of the vascular clamp at the time, was recorded. After repeating the measurements three times, the average value was calculated, as shown in Fig. 3.

**Figure 4:**
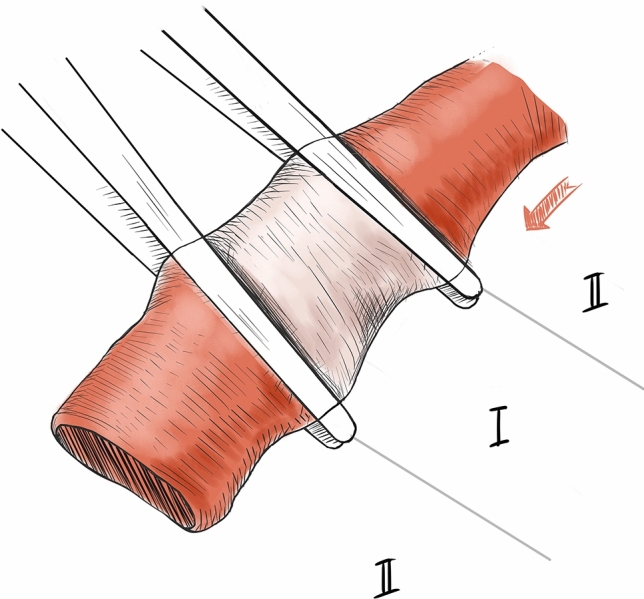
Schematic Diagram of Successful Blood Blocking. After the blood vessel was clamped proximally, microscopic tweezers were used to compress it and drain the blood out in a distal direction starting from the clamp. Failure of the evacuated part of the blood vessel to refill or leak was taken to indicate that blood flow had been blocked. (Adobe Photoshop: 21.0.1 20,191,106.r.47 2019/11/06:3152b481f18 × 64 https://www.adobe.com/cn/products/photoshop.html).

The MOF was determined as follows. First, evidence of successful blocking of a blood vessel was defined and the methods commonly used in microsurgery were adopted. After the blood vessel was clamped proximally, microscopic tweezers were used to compress it and drain the blood out in a distal direction starting from the clamp. Failure of the evacuated part of the blood vessel to refill or leak was taken to indicate that blood flow had been blocked (Fig. 4). The MOF that blocks blood flow could thus be determined.

### Statistical analysis

SPSS 20.0 software was used for statistical analysis. Measurement data are expressed as mean and standard deviation $(\overline{x} \pm s)$. Comparisons between groups were performed with *t*-tests, with α = 0.05 being considered to denote significant differences. Spearman rank correlation analysis was performed on the difference between MOF and ACF and the absolute value of the differences between MOF and ACF and vascular diameter and blood pressure, respectively.

## Results

### Results of mathematical analysis of vascular closure model

From the above, it can be seen that the blood pressure F1 on the vessel wall side is theoretically equal to the rebound force F2 after refilling. Our experiments verified this. The average blood pressure of the 24 SD rats was 112.38 ± 6.72 mmHg, the average diameter of their abdominal aortas was 1.38 mm, the average diameter of their abdominal aortas when empty was 1.11 mm, the average deformation of the aortas was 0.27 mm, the average thickness of the abdominal aortic walls was about 0.1 mm, and the width of the vascular clamp was 2 mm. Using formulas (1) and (2), we calculated that F1 = 0.065 N and F2 = 0.0635 N, that is, F1≈F2. Thus, the measurements we obtained in practice were consistent with our theory. F3 is the pressure of blood on the vessel wall when that vessel is not deformed. A decrease in blood vessel diameter may result in an increase in the local blood pressure. The local data required to confirm this are difficult to obtain. F3 = F is calculated according to the formula and model analysis. When the blood vessel is completely occluded, the two layers of blood vessel wall overlap and there is very little deformation. The muscle fibres of the abdominal aortas of SD rats are not very thick. Additionally, blood itself has a certain viscosity^17^. We found in our experiments that the sheared blood vessels were in a closed state when they were not rinsed clean. Hence, we ignored the force of F4 in this experiment. Therefore, the theoretical MOF is the sum of F1 and F3. Thus, theoretically F1 = F and F3 = F, as follows.

5 $$\begin{aligned} {\text{MOF}} & = 2 \times {\text{F}}1 \\ & = 2 \times \left( {1/2 \times\uppi \times {\text{D}} \times {\text{W}} \times {\text{BP}}} \right) \\ \end{aligned}$$

### Calculation of theoretical minimum occlusive force and results of measurement of actual minimum occlusive force in rats

The above equation enables calculation of the MOF for different blood pressures, clamp widths, and vessel diameters. In the present study, the vessel clamp width was uniform at 2 mm and we measured the actual minimum occlusion force (ACF) in 24 SD rats. These data are shown in Table 1.

**Table 1:** Summary of theoretical and actual minimum occlusive force in 24 rats.

	NO. 1	NO. 2	NO. 3	NO. 4	NO. 5	NO. 6	NO. 7	NO. 8	NO. 9	NO. 10	NO. 11	NO. 12
Width of vascular clamp (W) mm	2	2	2	2	2	2	2	2	2	2	2	2
Vascular diameter (D) mm	1.38	1.40	1.58	1.52	1.37	1.41	1.36	1.38	1.39	1.45	1.37	1.34
Blood pressure (BP) mmhg	104	92	89	91	88	64	84	60	82	88	74	84
Theoretical value (MOF) g	12.05	10.77	11.80	11.55	10.09	7.55	9.57	6.93	9.54	10.68	8.49	9.43
Actual value (ACF) g	11.50	11.00	12.00	11.20	10.20	8.20	8.80	8.20	10.50	10.50	8.60	9.20

	NO. 13	NO. 14	NO. 15	NO. 16	NO. 17	NO. 18	NO. 19	NO. 20	NO. 21	NO. 22	NO. 23	NO. 24
Width of vascular clamp (W) mm	2	2	2	2	2	2	2	2	2	2	2	2
Vascular diameter (D) mm	1.41	1.39	1.47	1.43	1.36	1.37	1.39	1.55	1.35	1.41	1.27	1.39
Blood pressure (BP) mmhg	98	82	89	94	90	61	78	84	98	84	64	77
Theoretical value (MOF) g	11.57	9.51	10.95	11.25	10.25	7.00	9.10	10.90	11.07	9.92	6.81	8.99
Actual value (ACF) g	9.80	9.20	10.50	9.80	9.80	7.20	9.60	10.50	10.40	9.60	7.40	8.60

※MOF_g_ = minimum occlusive force in grams.

ACF_g_ = actual occlusive force in grams.

The obtained data indicate that the theoretical MOF calculated by the formula is consistent with the actual MOF.

### Statistical analysis

SPSS 20.0 software was used to perform *t*-test analyses of the data. The calculated probability was 0.315, that is, the difference between the theoretical and actual MOF was not considered to be 0 at the level of α = 0.05 under the condition of normal distribution. Thus, the difference between these two values was not statistically significant. This is further evidence that the actual measured MOF tends to be consistent with the theoretical MOF calculated by the provided formula, which verifies the accuracy of the formula.

Spearman analysis showed that neither the difference between MOF and ACF nor the absolute value of that difference are related to the arterial diameter (P = 0.684, P = 0.780, P > 0.05). Additionally, Spearman analysis of blood pressure showed that the absolute value of the difference between the two is not related to blood pressure (P = 0.597, P > 0.05). However, the difference between MOF and ACF was positively correlated with blood pressure (r = 0.692, P < 0.001).

## Discussion

Our experiments on the abdominal aortas of SD rats verified the accuracy of the theoretical, mathematically predicted MOF of the vascular closure model. In addition, the model and formula have improved on the previous research of Dujovny et al.^11^ and Trobec and Gersak^12^. The advantages of our proposal include that the value of the calculated MOF is closer to the actual value and the variables involved in the formula are easier to measure. However, the vascular closure model we have established and the means of analysing the force are relatively simple and do not take all influencing factors into account. For example, the elasticity coefficient of closed blood vessels may change, systemic and local hormone regulation affects tissues during surgery, and blood is a non-Newtonian fluid. Additionally, there would have been some measurement errors during the experiment that may have affected the final results; however, the range of possible errors is relatively small.

The arteries of rats are not identical in structure and composition to those of humans. Because rats’ abdominal aortas are of smaller diameter and have a thinner middle layer of muscle fibre tissue in their walls, the severed ends of their aortas do not maintain the original shape of the lumen when the blood in them has been incompletely flushed out. Hence, we did not take the elastic force of this part of the blood vessel wall into account in the closed model.

The key to performing vascular anastomoses in microsurgery lies in the successful blocking of blood flow during the procedure and prevention of vascular crises postoperatively, which requires microsurgical instruments to be effective and safe and to cause minimal injury. Therefore, through experiments, we can provide reference data for clinical research and development and manufacture of medical instruments that cause minimal injury. In particular, vascular clamps that both safely prevent blood flow and cause minimal or no damage should be developed. Clinicians can minimise injury by selecting vascular clamps that are appropriate for each patient.

First, a relatively small total of 24 groups of valid statistical results was obtained in this experiment. In addition, these 24 sets of data were obtained randomly through our experiments; thus, random errors were inevitable. Second, the purpose of this experiment was to determine the accuracy of the improved formula, focusing on the absolute values of the differences between the obtained data. The smaller the absolute value of the difference between them, the closer the two actually are. According to *t*-tests, they did not differ significantly.

Further studies are needed to determine whether the differences between the two values are positively correlated with blood pressure. Our research group plans for this to be the focus of our next in-depth study.

Many types of vascular clamps are currently available, being made of different materials, capable of providing different occlusive forces, and having different surfaces. However, there are few reports on injury and healing after application of different vascular clamps. As a next step, we plan to research these matters.
